# Microtubule Motor Transport of Organelles in a Specialized Epithelium: The RPE

**DOI:** 10.3389/fcell.2022.852468

**Published:** 2022-03-04

**Authors:** Roni A. Hazim, David S. Williams

**Affiliations:** ^1^ Department of Ophthalmology and Stein Eye Institute, Los Angeles, CA, United States; ^2^ Department of Neurobiology, David Geffen School of Medicine at UCLA, Los Angeles, CA, United States; ^3^ Molecular Biology Institute, University of California, Los Angeles, Los Angeles, CA, United States; ^4^ Brain Research Institute, University of California, Los Angeles, Los Angeles, CA, United States

**Keywords:** RPE—retinal pigment epithelium, organelle transport, kinesin, dynein, retinal degeneration

## Abstract

The retinal pigment epithelium (RPE) is a uniquely polarized epithelium that lies adjacent to the photoreceptor cells in the retina, and is essential for photoreceptor function and viability. Two major motile organelles present in the RPE are the melanosomes, which are important for absorbing stray light, and phagosomes that result from the phagocytosis of the distal tips of the photoreceptor cilium, known as the photoreceptor outer segment (POS). These organelles are transported along microtubules, aligned with the apical-basal axis of the RPE. Although they undergo a directional migration, the organelles exhibit bidirectional movements, indicating both kinesin and dynein motor function in their transport. Apical melanosome localization requires dynein; it has been suggested that kinesin contribution might be complex with the involvement of more than one type of kinesin. POS phagosomes undergo bidirectional movements; roles of both plus- and minus-end directed motors appear to be important in the efficient degradation of phagosomes. This function is directly related to retinal health, with defects in motor proteins, or in the association of the phagosomes with the motors, resulting in retinal degenerative pathologies.

## Introduction

Motor-dependent intracellular transport is an essential process for all eukaryotic cells. It underlies numerous critical functions, including the trafficking and positioning of organelles, the endo- and exocytosis of biological molecules, the establishment of polarized domains, and the translocation of chromosomes during cell division. Impaired motor-dependent trafficking is at the root etiology of several neuro-pathologies, lysosomal storage disorders, and ciliopathies. *In vitro* models well suited for the study of intracellular transport, and its disease-related mechanisms, include polarized cells, such as neurons and epithelial cells, in which specific cargos must be trafficked to distinct cellular compartments.

The retinal pigment epithelium (RPE) is a polarized monolayer of cells situated between the light-sensitive photoreceptor cells and the nutrient-rich choriocapillaris (Lakkaraju et al., 2020). These pigmented cells perform supportive functions that are vital to the health of photoreceptors and the retina as a whole. Functions include: 1) absorption of stray light, 2) transport of water, ions, nutrients, and waste products, 3) recycling of the visual chromophore, 4) secretion of growth and signaling factors, 5) phagocytosis of distal photoreceptor outer segment (POS) disk membranes, and 6) contribution to the metabolic ecosystem of the outer retina (Strauss, 2005; Lakkaraju et al., 2020). In the mouse retina, a single RPE cell serves up to 200 photoreceptor cells (Volland et al., 2015). RPE cells are postmitotic and must therefore perform these functions throughout the lifetime of the organism (Ts’o and Friedman, 1967). The supportive capability of the RPE in the retina is heavily dependent on its differentiated state (Zhao et al., 2011), which represents a highly specialized epithelium (Lakkaraju et al., 2020).

Studies of the properties of specialized cells, like the RPE, tell us about the generality of basic principles and adaptions of molecular mechanisms. In this Mini Review, we highlight the role molecular motor proteins play in facilitating the functions of polarized RPE cells. We focus on microtubule motor-driven motility of two major RPE organelles: melanosomes, which characterize the RPE in most animals, and phagosomes, which result from the daily ingestion of POS tips. We also discuss how defective microtubule motor transport may contribute to retinal pathologies that impair our sense of vision.

### Organization of the Microtubule Cytoskeleton in the RPE

**FIGURE 1:**
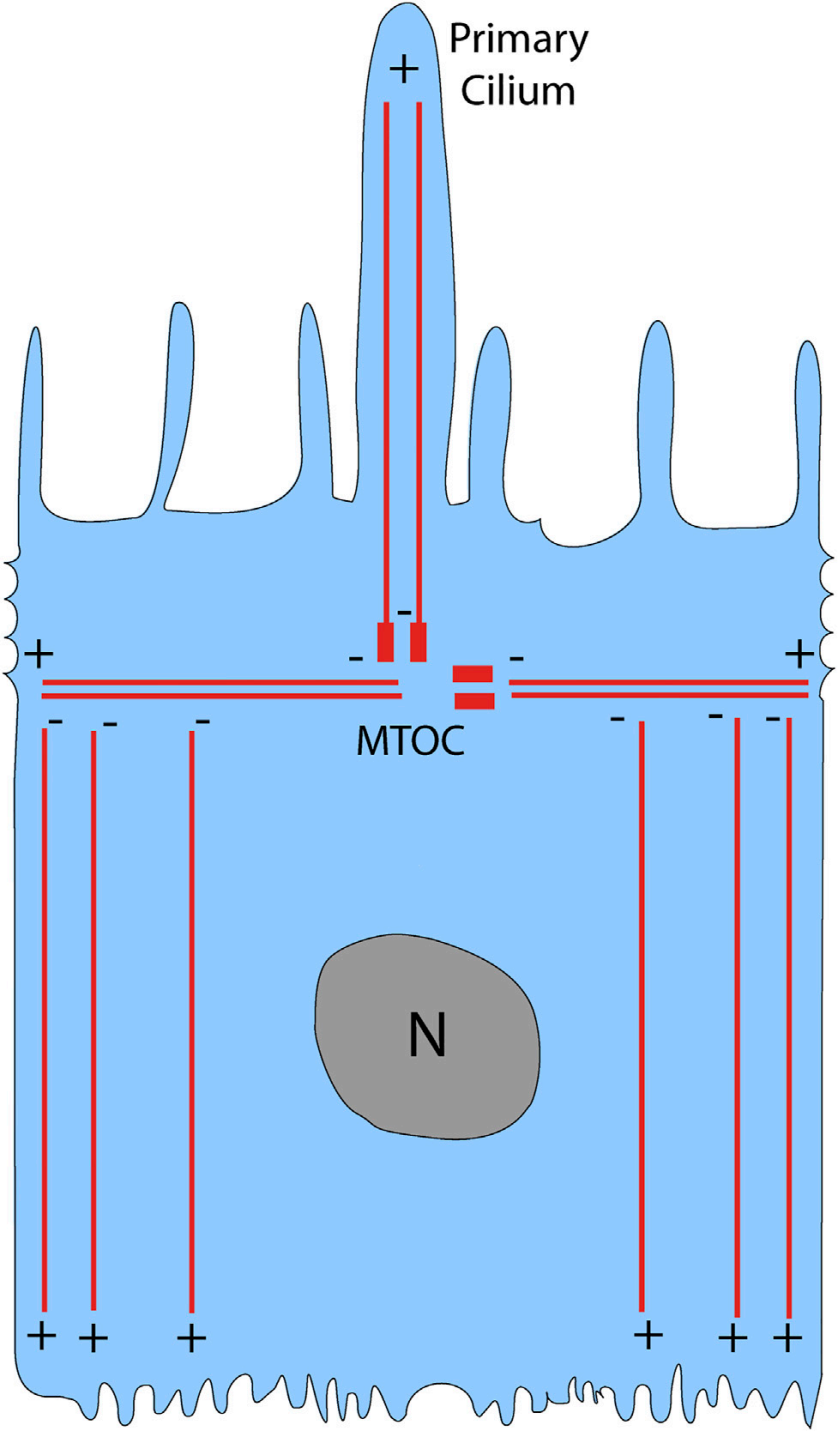
Microtubule organization in the RPE. In a polarized epithelial cell like the RPE, the microtubule-organizing center (MTOC) is located above the nucleus (N), where it gives rise to horizontal microtubules as well as vertical microtubules that emanate to the primary cilium. The majority of microtubules, however, are non-centrosomal vertical microtubules with their plus-ends located near the basal surface of the RPE. Modified from Jiang et al. (2015).

Cells have a cytoskeleton composed of microtubules, actin, and intermediate filaments, which maintain cell shape and organization. Of these cytoskeletal elements, the microtubules are the biggest; they are composed of *a*- and *ß*-tubulin monomers that form a hollow cylindrical polymer 25 nm in diameter (Akhmanova and Steinmetz, 2008). Microtubules are highly dynamic and polarized structures, with a plus-end that is fast growing and a minus-end that is slow growing (Mitchison and Kirschner, 1984). In a nonpolarized cell, the microtubule plus-end is directed towards the cell periphery while the minus-end is anchored at the microtubule-organizing center (MTOC) (Vorobjev and Nadezhdina, 1987). The MTOC is composed of the centrosome and surrounding pericentriolar material, and typically lies near the nucleus (Vorobjev and Nadezhdina, 1987). In contrast, polarized cells such as the RPE have an MTOC that has migrated away from the nucleus and towards the apical region of the cell (Bacallao et al., 1989; Bre et al., 1990). The microtubule organization of the RPE is similar to that reported for other confluent epithelial cells (Bacallao et al., 1989; Gilbert et al., 1991; Jiang et al., 2015). Horizontal microtubules emanate from the MTOC, which is at the level of the circumferential actin filaments (Jiang et al., 2015), and are perpendicular to the apical-basal axis of the cell (Figure 1). The majority of microtubules have their minus-ends at the level of the MTOC, but they are removed from the MTOC and are aligned vertically along the apical-basal axis of the cell; their plus-ends face the basal surface (Musch, 2004; Jiang et al., 2015) (Figure 1). Thus, the apical region of the cell is devoid of microtubules, except for those in the cilium, which extends from the apical surface; actin filaments occupy the entire apical region, including the apical microvilli.

In both polarized and nonpolarized cells, the microtubules serve as tracks for molecular motors that transport a variety of cargos (Toomre et al., 1999). Two families of motor proteins travel along microtubules. Kinesins are plus-end-directed motors while dyneins are minus-end-directed motors. The cargos transported by these two motors include, membrane-bound organelles, proteins, mRNA, and viruses (Cianfrocco et al., 2015). The kinesin superfamily of motors is quite diverse, with 15 identified families. Even with such diversity, kinesins require adaptor proteins that ensure specificity of cargo binding (Tempes et al., 2020). In contrast to the large kinesin family, dyneins are based on far fewer different motor subunits (heavy chains). However, these motor subunits associate with a large variety of other subunits, as well as adaptor proteins that are essential for cargo specificity and linkage to dynein in the cytoplasm of animal cells (Reck-Peterson et al., 2018). Moreover, the adaptor proteins allow the dynein motor to travel with processivity such that it does not detach from microtubules before reaching its destination (McKenney et al., 2014; Schlager et al., 2014). In the RPE, microtubule motors transport two important motile organelles necessary for their supportive function, including the pigment-containing melanosome and the POS-derived phagosome.

### Transport of Melanosomes in the RPE

Melanosomes play an important role in the absorption of stray light in the eyes of invertebrates and vertebrates, with their movements providing a means to alter visual sensitivity and resolution. Examples are found among mollusks (Daw and Pearlman, 1974), arthropods (Boschek, 1971; Williams, 1982; 1983), and fish and amphibians (Back et al., 1965; Burnside and Basinger, 1983; Burnside, 2001; Burgoyne et al., 2015). In vertebrate eyes, cylindrically-shaped melanosomes enter the narrow apical processes of the RPE, which project among the POS, thus affecting light absorption by the POS. In addition to screening light, melanosomes may also contribute to the heavy phagocytic role of the RPE, as they contain proteases (Azarian et al., 2006), and have been observed to fused with phagosomes (Schraermeyer et al., 1999).

**FIGURE 2:**
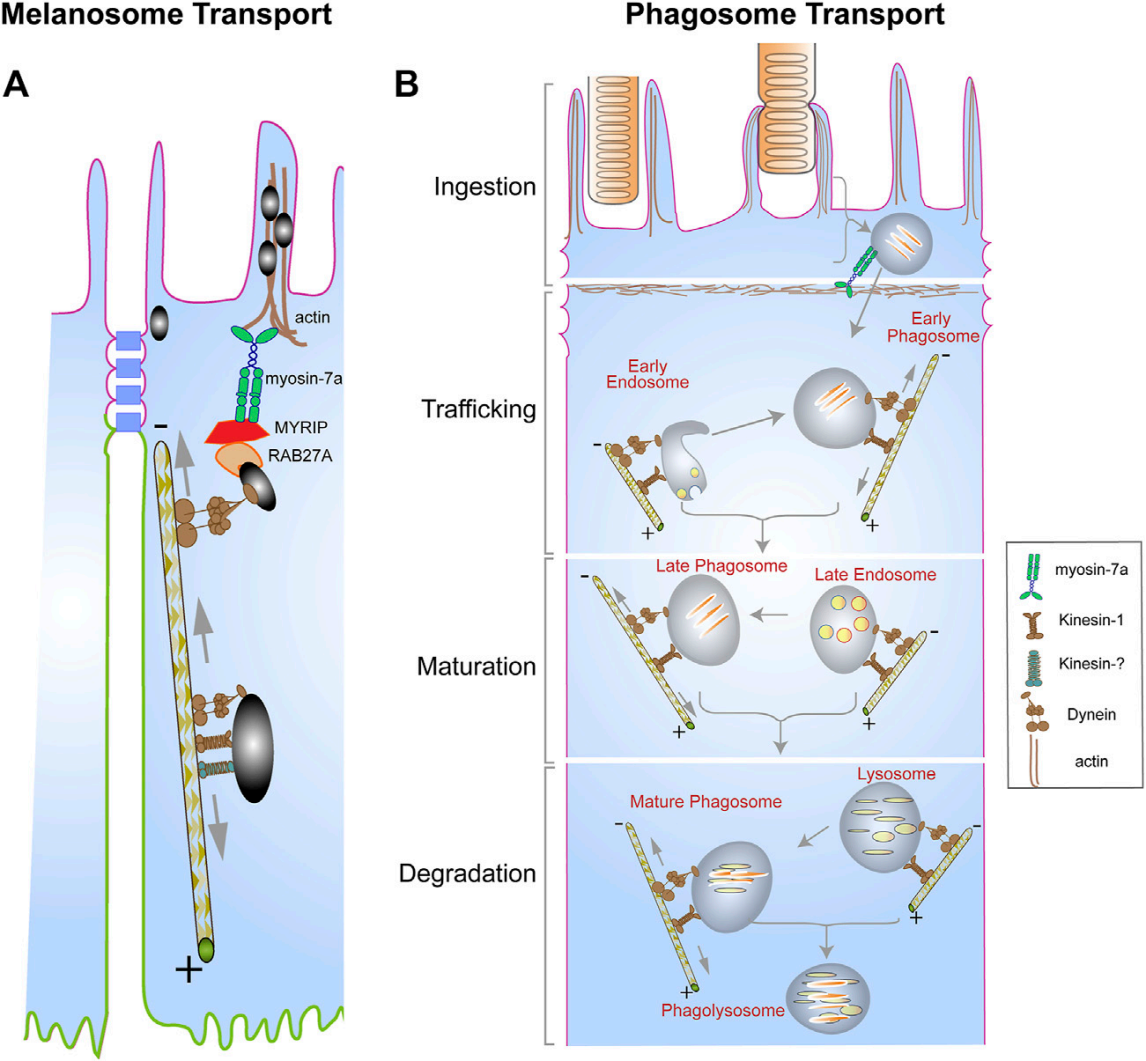
Organelle transport in the RPE. **(A)** Pigment-containing melanosomes found in the RPE cell body exhibit bidirectional movements on microtubules due to the opposing actions of dynein, and kinesin-1, plus possibly a second different kinesin. The trafficking of the melanosomes by dynein towards the apical region is necessary for their capture by the actin-based motor, myosin-7a, in complex with RAB27A and MYRIP, resulting in their localization in the actin-rich apical region of the RPE. **(B)** Photoreceptor outer segment (POS) disk membranes ingested by the RPE form a motile organelle, the phagosome, which undergoes a series of interactions with endosomes (early and late) and lysosomes to ensure timely degradation and clearance. These interactions are facilitated by the opposing actions of dynein and kinesin motors, which mediate the bidirectional movement of the phagosomes, endosomes, and the lysosomes. Although the kinesin and dynein motors appear far apart on a particular organelle in both **(A,B)**, they may be in a complex that associates with the cargo *via* shared adaptor proteins (Fenton et al., 2021). Several alternative mechanisms have been proposed to drive bidirectional movements of organelles, including coordination between the kinesin and dynein motors that is regulated by adaptor proteins, or, more simply, a tug-of-war between the two types of motors (Encalada and Goldstein, 2014). Modified from original drawings, prepared by Aparna Lakkaraju and published in Lakkaraju et al. (2020).

The localization of melanosomes to the actin-rich apical RPE is dependent on the actin-based motor protein, myosin-7a, which functionally associates with RPE melanosomes by linkage through RAB27A and the exophilin, MYRIP (El-Amraoui et al., 2002; Futter et al., 2004; Gibbs et al., 2004; Klomp et al., 2007; Lopes et al., 2007). This apical localization also requires cytoplasmic dynein, with lack of cytoplasmic dynein or myosin-7a resulting in comparable phenotypes (Liu et al., 1998; Jiang et al., 2020). It appears that dynein is responsible for delivering melanosomes from the cell body to the apical region of the RPE, and myosin-7a takes delivery and keeps them in the actin-rich, apical domain. However, in the cell body, melanosomes undergo bidirectional movements along microtubules (Jiang et al., 2020), suggesting the involvement of a kinesin as well as dynein (Figure 2A). Loss of kinesin-1 function (due to loss of KIF5B) affected melanosome motility, although, unlike loss of cytoplasmic dynein function, it did not have an obvious effect on overall melanosome localization (Jiang et al., 2020). In some instances, plus-end directed microtubule movement involves more than one kinesin (Shin et al., 2003; Miller et al., 2005; Twelvetrees et al., 2010; Nakajima et al., 2012). Perhaps loss of kinesin-1 function, together with the inhibition of a second kinesin, would completely inhibit plus-end directed movements, and thus have an overall effect on melanosome localization within the RPE.

Melanosome transport in the RPE can be compared to that in skin melanocytes, but there are significant distinctions. In melanocytes, there is a net migration of melanosomes from the cell body to the dendrites, in the periphery of the cell; from there they are delivered to the keratinocytes (Wasmeier et al., 2008; Hume and Seabra, 2011; Wu and Hammer, 2014; Moreiras et al., 2019). Melanosome movements along the microtubules are nevertheless bidirectional, with transport towards the nucleus driven by dynein (Byers et al., 2000). Their movement to the dendrites was proposed to be driven mainly by kinesin-1 (Ishida et al., 2012; Ishida et al., 2015). However, more recent evidence argues that kinesin-1 is not necessary for movement to the dendrites, and that it can be accomplished entirely by myosin-5a activity along cortical actin filaments (Evans et al., 2014; Robinson et al., 2017).

The dynein-driven motility of melanocyte melanosomes is an example of where dynein adaptor proteins have been reported to function as regulatory linkers between motor and cargo. The adaptors include melanoregulin (Ohbayashi et al., 2012) and Rab36 (Matsui et al., 2012). Interestingly, melanoregulin in the RPE appears to be involved in the maturation of POS-derived phagosomes rather than melanosome transport (Damek-Poprawa et al., 2009). Further studies are necessary to reveal the mechanisms by which melanosomes link to microtubule motors of the dynein and kinesin family.

### Transport of Phagosomes in the RPE

The RPE plays an essential role in the renewal of the outer segments of photoreceptor cells (Young and Bok, 1969; Williams and Fisher, 1987). The outer segments contain thousands of lipid disk membranes, the site where phototransduction begins (Burns and Arshavsky, 2005). New disk membranes are continually synthesized and formed at the base of the POS (Young, 1967). The apical surface of the RPE faces the photoreceptor cells, with its apical microvilli interdigitating the POS. Following a circadian rhythm, the RPE phagocytizes the tips of the POS. The phagosome containing these lipid disk membranes is unique in its composition, and appears to undergo a series of interactions to be degraded properly (Bosch et al., 1993; Wavre-Shapton et al., 2014).

The processing of POS-derived phagosomes in the RPE is heavily dependent on their motor-dependent trafficking to ensure interactions with degradative organelles, including endosomes and lysosomes (Figure 2B). Studies by Herman and Steinberg showed that disruption of the microtubule cytoskeleton in tapetal RPE of the opossum impedes the movement of POS-derived phagosomes (Herman and Steinberg, 1982b; a). More recently, the association of POS-derived phagosomes with a light chain of the kinesin-1 motor (KLC1) was observed as the phagosomes transitioned from the apical region to the cell body of mouse RPE (Jiang et al., 2015). Lack of KLC1 in mouse RPE impaired the trafficking of the phagosomes towards the basal surface of the RPE as well as their degradation (Jiang et al., 2015). Interestingly, live-cell imaging of POS-derived phagosomes in primary mouse RPE cells has shown that these organelles exhibit bidirectional movements along the microtubules (Jiang et al., 2015; Hazim et al., 2016), suggesting the actions of opposing motors; although the exact role of the minus-end dynein motor in the transport of POS-derived phagosomes remains unclear. The bidirectional movement of phagosomes may be necessary to ensure sufficient interactions with early and late endosomes, which prime the phagosome for fusion with lysosomes. Bidirectional movements of phagosomes, particularly in combination with bidirectional movements of endolysosomes on the same or nearby microtubules, should increase the probability of interactions between phagosomes and endolysosomes. Presumably, the number of such interactions is related to the efficiency of phagosome maturation and degradation—and whether undigested lipid membranes accumulate (Keeling et al., 2019).

Although there is evidence that microtubule-based motor transport is essential for the proper trafficking of POS-derived phagosomes, and therefore their timely clearance, there is still a knowledge gap with respect to the transport mechanisms that underlie this process. This gap includes the mechanisms that regulate the bidirectional motility of the phagosome, as well as the identification of adaptor proteins that facilitate the recruitment of motor proteins to the phagosome membrane.

### Motor Proteins in the Context of RPE and Retinal Pathology

The RPE is essential to retinal health. Most forms of macular degeneration, including age-related macular degeneration (AMD), a common affliction among the elderly (Wong et al., 2014), arise from primary lesions in the RPE. Several monogenic forms of retinal degeneration are also caused by mutations in genes that are expressed in the RPE; for example, mutations in *MERTK* (Gal et al., 2000), which cause retinitis pigmentosa, *CHM* (Cremers et al., 1990), which cause choroideremia, and *MYO7A* which cause Usher syndrome type 1B (Weil et al., 1995). Studies from animal models provide some insight into whether defects in the motility or localization of organelles transported by microtubule motors could contribute to RPE dysfunction and pathology.

Melanosomes in mouse models of choroideremia (Tolmachova et al., 2010) and Usher syndrome 1B (Liu et al., 1998) do not localize normally to the apical region, but these models also demonstrate other potentially more serious RPE defects, including retarded POS phagosome degradation (see below) (Gibbs et al., 2003; Gordiyenko et al., 2010; Lopes et al., 2011; Williams and Lopes, 2011; Wavre-Shapton et al., 2013). Whether this mislocalization contributes to retinal pathology is not known. Nevertheless, it seems that melanosome mislocalization could be a useful biomarker for diseases such as choroideremia and Usher syndrome 1B, since it may be detectable with advanced retinal imaging (Liu et al., 2019).

Given how important the trafficking of phagosomes is to their timely processing and clearance, it is perhaps no surprise that motility defects result in pathological conditions that affect the health of the RPE and therefore the retina. In mice lacking kinesin-1 light chain, phagosome degradation was delayed resulting in AMD-like symptoms in aged animals, including oxidative stress, complement system activation, and photoreceptor death (Jiang et al., 2015). Interestingly, a similar pathology was reported in mice with RPE-specific ablation of the orthologue of the *CHM* gene, which encodes Rab Escort Protein-1 (REP-1) (Wavre-Shapton et al., 2013). REP-1 functions in the prenylation of Rab proteins, which serve as regulators of membrane trafficking. REP-1 depletion in human fetal RPE has been shown to impair the association of POS-derived phagosomes with RAB7-positive late endosomes, thereby delaying their clearance by the RPE (Gordiyenko et al., 2010).

Defects in dynein-specific trafficking of phagosomes have also been shown to impair the clearance of these organelles by the RPE. In a mouse model of Stargardt 3 macular degeneration, phagosomes contain a mutant form of the elongation of very long-chain fatty acids 4 (ELOVL4) protein, and were found to exhibit increased association with dynein and reduced clearance (Esteve-Rudd et al., 2018). This study indicates that the content of the POS-derived phagosome is an important characteristic that affects the recruitment and association of motor proteins with organelles in the cell. With artificial phagosomes, generated by the phagocytosis of latex beads, the cholesterol content of the enclosing membrane has been shown to influence phagosome motility by the dynein motor (Rai et al., 2016). Targeted approaches to modify the POS content prior to RPE phagocytosis would provide a physiological system for testing how cholesterol, and other macromolecules, affect the trafficking and clearance of phagosomes. Elucidating these mechanisms would provide a better understanding of how motor dysfunction and impaired subcellular trafficking contribute to pathology in the RPE and the retina.
